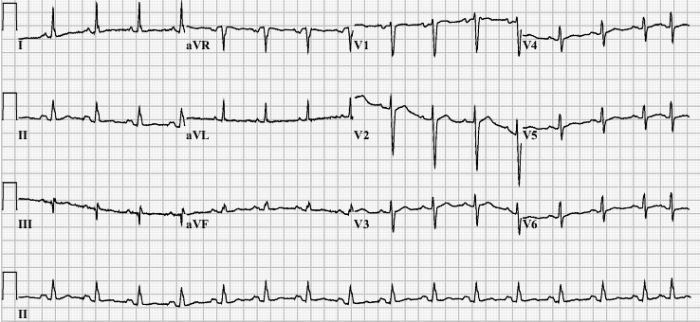# ECG For The Diagnosis Of Pulmonary Embolism When Conventional Imaging Cannot Be Utilized: A Case Report And Review Of The Literature

**Published:** 2009-09-01

**Authors:** Keith Todd, Christopher S Simpson, Damian P Redfearn, Hoshiar Abdollah, Adrian Baranchuk

**Affiliations:** Division of Cardiology, Department of Medicine, Kingston General Hospital, Queen's University, Kingston, Ontario, Canada

**Keywords:** Pulmonary Embolism, ECG

## Abstract

The diagnosis of acute pulmonary embolism has always been challenging.  However, it has recently been greatly assisted through advances in radiological imaging. While imaging techniques are widely available, they cannot always be utilized. We report a case of acute pulmonary embolism in a patient with several prior pulmonary resections that would likely result in a non-diagnostic V/Q scan and acute renal insufficiency that was a relative contraindication to CT pulmonary angiography. The patient's electrocardiogram displayed several features suggestive of acute pulmonary embolism, which in the absence of effective radiological imaging, were essential in her diagnosis and management.

## Introduction

Interest in diagnosis of acute pulmonary embolism (PE) utilizing the electrocardiogram (ECG) has decreased since the creation of imaging techniques such as V/Q scanning and CT pulmonary angiography. While these techniques provide superior sensitivity and specificity to diagnose PE, they cannot always be utilized. A small but significant number of patients have co-morbid conditions that make these imaging techniques either contraindicated or non-diagnostic. In these circumstances, the ECG in addition to clinical acumen can be essential in directing the physician towards the diagnosis. While no isolated ECG abnormality is definitively associated with PE, certain constellations of ECG abnormalities have been shown to be reasonably specific [1-3]. In this report, we describe a case where ECG abnormalities were essential in the initial diagnosis and management of acute PE, as imaging techniques were either contraindicated or likely to be non-diagnostic.

## Case

A 64-year-old female with a remote history of right lower lobectomy for stage 1B non-small cell lung cancer presents with new onset confusion three weeks post excision of a left upper lobe lung nodule, subsequently determined to be metastases from her right lung. The confusion began one week after discharge and progressed over a two-week period prior to presentation, she had no prior history of cognitive dysfunction.

On initial assessment, the patient was agitated and confused and thus unable to provide a detailed history. Her current medications were; bisoprolol, methotrimeprazine, glatiramer acetate, trazodone, venlafaxine, controlled release morphine sulfate, lorazepam and dicolfenac/misoprostol. Physical examination showed vital signs as follows; heart rate 63 bpm, blood pressure 101/62mmHg, temperature 36º C, respiratory rate 24 and SpO_2_ 94% on 5L 0_2_. Cardiac exam revealed a normal S1, loud S2, no S3 or S4 and a I/IV holosystolic murmur that was more pronounced with inspiration. The meniscus of the JVP was not visible, however it was occludable, suggesting its level was above the angle of the jaw with the patient upright. No heaves, pedal edema, or pulsus paradoxus were observed. The lungs were clear with decreased air entry over the right lung base and left upper lobe consistent with prior lung resections. No focal neurological deficits were apparent and no other abnormalities were noted.

Laboratory values revealed acute renal insufficiency with serum creatinine of 149 µmol/L, up from baseline of 75µmol/L three weeks previously. Creatinine kinase and troponin I were within normal range, 84 and 0.055 respectively. The D-dimer was markedly elevated, > 4.04ug FEU/ml. Blood glucose, white blood cell count and extended electrolytes were all within normal limits.  Venous blood gas revealed pH 7.30, pO_2_ 29.3, pCO_2_ 52.7, HCO_3_ 25 on 5L 0_2_. The respiratory acidosis and hypercarbia was attributed to her underlying COPD and reduced clearance of opiod metabolites, the slight HCO_3_ elevation to volume contraction. The chest x-ray was not significantly changed from previous and a CT scan of the head did not reveal any acute intracranial abnormality.

**Figure 1:**
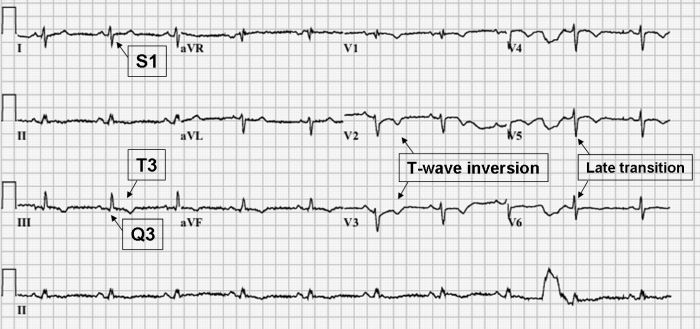


Examination of the admission 12-lead ECG revealed: incomplete right bundle branch block (RBBB), right axis deviation (RAD), S_1_Q_3_T_3_ pattern, T-wave inversion in leads V1-V6 and a late transition in the precordial leads (Figure 1).

The clinical presentation of hypoxia and relative hypotension on a background of malignancy, recent surgery and significant ECG changes were quite concerning for possible PE. The patient's confusion, while not typical of acute PE, has been recognized in the historical literature, with up to 5% displaying some form of neurological manifestation [4]. In this case the confusion was likely multifactorial being a product of hypoxia, hypercarbia and reduced renal clearance of her medications.

After initial stabilization, most treatment algorithms would advocate some form of imaging to confirm the diagnosis, usually a V/Q scan or CT pulmonary angiogram (CTPA). However, it was not possible to arrange a prompt V/Q scan, and given our patient's prior lung resections the result would likely be non-diagnostic. A CTPA would have been preferable but her acute renal insufficiency was a relative contraindication.

The use of lower limb Doppler ultrasound to help predict acute PE was considered, however the evidence to support its use is controversial, with some studies demonstrating as few as 29% of patients with confirmed PE having evidence of DVT [5]. A transesophageal echo would have been technically very difficult in this patient given her oxygen requirements and degree of agitation. A transthoracic echocardiogram (TTE) may have provided some indirect evidence of PE including signs of RV dysfunction and/or pulmonary hypertension, however, we already had strong ECG evidence for this and felt TTE would provide limited additional information.

Based on a high degree of clinical suspicion and significant ECG findings the patient was started on empiric anti-coagulation. She showed substantial improvement in her oxygen requirements and hemodynamic stability over the following 24 hour and the diagnosis of acute PE was confirmed by CTPA two days later after the resolution of her acute renal insufficiency.

## Discussion

More than seven decades ago, McGinn and White described the first association between acute PE and specific ECG changes when they noted the familiarS_1_Q_3_T_3_ pattern in 7 patients with acute cor pulmonale [6].  Numerous articles have been published since then describing the association between various ECG patterns and the diagnosis, severity and/or outcome of acute PE. Several of the more frequently described associations include: normal ECG, sinus tachycardia, complete and incomplete RBBB, axis changes, transition zone shift, low voltage, ST-segment and T-wave changes, S_1_Q_3_T_3_ pattern, P-pulmonale and atrial arrhythmias [7,8].

The following is a brief review of the possible ECG changes associated with acute PE:

### Normal ECG

A normal ECG has been reported in many studies of patients presenting with acute PE, with an incidence ranging from 9-30% [7-11].  Sreeram et al reported that the majority of patients presenting with a normal ECG maintained the rhythm throughout their hospitalization [2].

### Sinus tachycardia

Sinus tachycardia is an abnormality frequently associated with acute PE, with a reported incidence of between 8%-69% [7].

### Right Bundle Branch Block (RBBB)

Incomplete or complete RBBB has been associated with acute PE in a number of studies with variable incidence ranging from 6%-67% [7,8]. While this finding is felt to be fairly typical, a review by Ullman et al, estimated the overall incidence to be only 25% [8]. As well, the finding of PE associated RBBB is often transient in nature resolving within 3 months to 3 years [12]. Sorokina attributes the finding of RBBB to acute right ventricular overload and dilation, accompanied by subendocardial ischemia in the right bundle [13,14]. The appearance of a RBBB pattern has been noted to be more frequent in cases of massive trunk obstruction than peripheral embolism [14]. This is likely a result of its greater potential to produce acute right ventricular overload.

### Axis Changes

LAD, RAD and indeterminate QRS axis changes have all been associated with acute PE. While RAD has often been described as the 'classic' axis deviation, LAD is observed more frequently [7,8].  However, this observation may be influenced by individuals with pre-existing cardiopulmonary disease. In the Urokinase-Pulmonary Embolism Trial (UPET) LAD was reported more frequently than RAD, however, when individuals with pre-existing cardiopulmonary disease were excluded, the incidence was equivalent [8,15].

### Transition Zone Shift

The precordial lead in which the magnitude of R and S-waves become equivalent normally occurs at leads V3 or V4 and is often referred to as the 'transition zone'. A shift in the transition zone leftward so that the R and S-waves are equivalent at leads V5 or beyond has been reported by a number of studies [7,16] with an incidence of up to 51% reported in one study [2]. The cause of the leftward shift of the transition zone is not completely understood but has been postulated to be the result of biochemical abnormalities and hypoxia of the myocardium and His-Purkinje system slowing conduction in the RV, as well as through RV dilation itself [16]. RV dilation contributes to QRS axis shifts both anatomically and electrically, by physically altering the position of the heart in relation to the cardiac leads and by variably slowing conduction in the right bundle and RV such that the RV is responsible for greater proportion of the terminal depolarization vectors in the QRS complex. Thus, the QRS axis tends to shift rightward with a resultant left shift of the precordial transition zone.

### Low Voltage

Several studies have noted low voltages, defined as greatest overall deflection of the QRS complex ≤ 5mm in all limb leads, to be associated with acute PE [1-3,9,15,17]. While the finding is frequently noted, it is quite variable with an incidence of 6-30% [3,10].

### ST-segment and T-wave changes

ST-segment and T-wave changes are the most frequently noted abnormality associated with acute PE [2,7,15,18]. Non-specific ST-segment elevation and depression has been reported in up to 49% of all patients with acute PE [11]. In patients where the diagnosis of PE has been confirmed, some studies have used T-wave inversion as a diagnostic tool for predicting the severity of acute PE and likelihood of complicated course [9,19,20]. Kosuge et al showed that the rate of RV dysfunction correlated with the number of leads displaying T wave inversion [19]. In this study, all patients with T wave inversion in ≥ 7 leads had evidence of RV dysfunction on echocardiography. This was also found to be an independent predictor of in-hospital complicated events including: need for CPR, catecholamine or mechanical cardiovascular support due to hemodynamic instability.

### S_1_Q_3_T_3_ pattern

This 'classic' pattern is often considered the pathognomonic ECG abnormality associated with acute pulmonary embolism. However, its reported incidence in acute PE is quite variable from 10-50% and in some studies has been found to be equally likely in patients without PE [1,7].

### P-pulmonale

P-pulmonale,  defined as P-wave amplitude greater than 2.5 mV in lead II, has been associated with right atrial enlargement and/or hypertrophy secondary to acute PE [2,15]. It has been reported with variable frequency from 2 to 31% of patients with acute PE [7].

### Atrial arrhythmias

A number of atrial arrhythmias can manifest from acute PE, these are thought to be related to increased atrial pressures and atrial enlargement [8]. Atrial fibrillation and flutter have been reported in up to 18% and 35% of acute PE patients, respectively [7]. However, other studies have reported the incidence to be as low as 0-5% [1,7,21].

Many of the ECG abnormalities associated with acute PE are thought to be the result of a right-sided heart strain pattern as well as possible myocardial ischemia at the cellular level [7]. Decreased perfusion within the lung secondary to the embolus is thought to cause a release of vasoconstrictive mediators such as serotonin and catecholamines [8,12,22]. Vasoconstriction along with mechanical obstruction from the thrombus, results in increased pulmonary artery pressures transmitted to the right heart. This increases right heart myocardial wall tension and can result in dilation of the right ventricle and right atrium, eventually resulting in contractile dysfunction and circulatory collapse.

As early as 1938, investigators attempted to explain the physiologic mechanism by which acute PE produces ECG abnormalities. Love et al. performed a series of experiments in which they mechanically obstructed the pulmonary artery (PA) producing a number of ECG changes similar to those found in clinical PE [21,23]. They noted that the ECG changes were ubiquitously preceded by RV dilation, leading them to conclude that RV dilation was responsible. While several other investigators [13,14,16] have drawn similar conclusions, it is unlikely to be the sole mechanism. Observations from other studies [2,9] have shown that RV dilation does not uniformly produce ECG abnormalities and, if they occur, there can be a significant lag time between initial RV dilation and onset of ECG abnormalities. As well, these abnormalities may persist long after the resolution of RV dilation and elevated pulmonary pressures making it difficult to suggest an isolated causal relationship [21].  As such, the physiologic explanation of the many ECG abnormalities associated with acute PE remains uncertain.

Much of the literature investigating the diagnosis of PE has focused on the limited sensitivity of the ECG. This has led many to suggest that it has little utility as a diagnostic screening tool for acute PE [7,8,10]. However, the usefulness of the ECG is not in its ability to identify all cases of PE, but in its ability to be reasonably specific given the appropriate ECG findings and clinical scenario.

While it has not been the focus of a clinical trial, it is reasonable to suggest that the specificity of the ECG is greatest when multiple abnormalities associated with PE are present concurrently and occur acutely. For instance, there are a number of disease processes that may produce a right-sided heart strain pattern over the course of months to years but there are few other than PE that are capable of doing so over the course of hours to weeks.

There have been several studies that have shown the ECG to have a high specificity to predict pulmonary hypertension and/or RV strain secondary to PE. Sreeram et al. attempted to develop prediction rules in order to aid in the ECG diagnosis of PE by identifying several features suggestive of RV strain  [2]. Forty-nine patients with proven PE and without previous lung disease were studied. Pulmonary embolism was considered probable (76%) if 3 or more of the following features were present:

1. Incomplete or complete RBBB, which was associated with ST-segment elevation and positive T-wave
2. S-wave in leads I and aVL >1.5mm
3. A shift in the transition zone in the precordial leads to V5
4. Q-wave in leads III and aVF, but not in lead II
5. Right-axis deviation, with a frontal QRS axis
6. A low voltage QRS complex < 5 mm in the limb leads
7. T-wave inversion in leads III and aVF or leads V1 to V4

The sensitivity of Sreeram's predictive rule to detect PE has not been validated by other studies, however they have shown it to be quite specific (94.2%)  [1]. A study by Punukollu et al showed that T-wave inversion in leads V1 to V3 had a specificity of 88% and diagnostic accuracy of 81% for RV dysfunction in acute PE  [24].  Daniel et al. developed an ECG scoring system based on typical features of acute PE such as presence of RBBB, T-wave inversions, and additional features of right heart strain that predicted severe pulmonary hypertension (sPAP >  50mm Hg) with a specificity of 97.7% if the patient's 'ECG score' ≥ 10  [20].  A study by Sukhija et al analyzed the prevalence of 18 ECG abnormalities in PE  [25]. A logisitic regression analysis from the study showed that the presence of 2 of the following 5 ECG abnormalities (S_1_, Q_3_, S_1_Q_3_T_3_ pattern, sinus tachycardia or supraventricular tachyarrhythmias) was associated with a specificity of 96% and a positive predictive value of 94%.

Comparison of our patient's admission ECG (Figure 1) with one obtained three weeks earlier, post surgical resection (Figure 2) shows many characteristic changes outlined by Sreeram  [2].  These include: new incomplete right bundle branch block (RBBB), right axis deviation (RAD), S1Q3T3, T wave inversion in leads V1-V6 and transition zone shift to V5. This constellation of ECG changes also produces an ECG score ≥  10 as outlined by Daniel et al and meets the criteria outlined by Sukhija et al producing specificities of 97.7% and 96% respectively, and a PPV of 94%  [20,25]. This number of ECG changes consistent with RV strain occurring over a 3-week period would be difficult to explain by any physiological mechanism other than acute PE.  Given the likelihood that a V/Q scan would be non-diagnostic and that we were initially unable to perform a CT pulmonary angiogram secondary to our patient's renal insufficiency, the application of Sreeram's predictive rule, Daniel's ECG score and Sukhija's regression analysis was important in raising our patient's pre-test probability of PE. On the basis of her clinical presentation and characteristic ECG changes we justified the use of anticoagulation. Several days later her renal insufficiency resolved and a CTPA confirmed the diagnosis of acute PE.

## Conclusion

The diagnosis of acute pulmonary embolism has always been challenging and while imaging techniques have markedly improved our diagnostic accuracy, they cannot always be utilized. This case provides an example of how the ECG is still relevant, and in certain circumstances essential, to the diagnosis of acute pulmonary embolism.

## Figures and Tables

**Figure 2:**